# Structural Elucidation and Engineering of a Bacterial Carbohydrate Oxidase

**DOI:** 10.1021/acs.biochem.2c00307

**Published:** 2022-07-26

**Authors:** Alessandro Boverio, Wahyu S. Widodo, Lars L. Santema, Henriëtte J. Rozeboom, Ruite Xiang, Víctor Guallar, Andrea Mattevi, Marco W. Fraaije

**Affiliations:** † Molecular Enzymology, Groningen Biomolecular Sciences and Biotechnology Institute, University of Groningen, 9747AG Groningen, The Netherlands; ‡ Department of Biology and Biotechnology, University of Pavia, via Ferrata 9, 27100 Pavia, Italy; § Electronic and Atomic Protein Modelling Group, Barcelona Supercomputing Center, E-08034 Barcelona, Spain

## Abstract

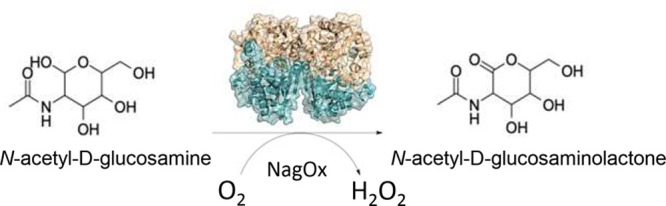

Flavin-dependent carbohydrate oxidases are valuable tools in biotechnological applications due to their high selectivity in the oxidation of carbohydrates. In this study, we report the biochemical and structural characterization of a recently discovered carbohydrate oxidase from the bacterium *Ralstonia solanacearum*, which is a member of the vanillyl alcohol oxidase flavoprotein family. Due to its exceptionally high activity toward *N*-acetyl-d-galactosamine and *N*-acetyl-d-glucosamine, the enzyme was named *N*-acetyl-glucosamine oxidase (NagOx). In contrast to most known (fungal) carbohydrate oxidases, NagOx could be overexpressed in a bacterial host, which facilitated detailed biochemical and enzyme engineering studies. Steady state kinetic analyses revealed that non-acetylated hexoses were also accepted as substrates albeit with lower efficiency. Upon determination of the crystal structure, structural insights into NagOx were obtained. A large cavity containing a bicovalently bound FAD, tethered via histidyl and cysteinyl linkages, was observed. Substrate docking highlighted how a single residue (Leu251) plays a key role in the accommodation of N-acetylated sugars in the active site. Upon replacement of Leu251 (L251R mutant), an enzyme variant was generated with a drastically modified substrate acceptance profile, tuned toward non-N-acetylated monosaccharides and disaccharides. Furthermore, the activity toward bulkier substrates such as the trisaccharide maltotriose was introduced by this mutation. Due to its advantage of being overexpressed in a bacterial host, NagOx can be considered a promising alternative engineerable biocatalyst for selective oxidation of monosaccharides and oligosaccharides.

Flavin-dependent carbohydrate oxidases make up a growing class of enzymes that can be subdivided into two major flavoprotein families depending on their structural fold: the glucose-methanol-choline (GMC) family and the vanillyl alcohol oxidase (VAO) family.^1−4^ Flavoprotein oxidases acting on carbohydrates represent highly valuable tools in biotechnology due to their ability to be highly regioselective and efficient in the oxidation of sugars. The most used carbohydrate oxidase is glucose oxidase (EC 1.1.3.4)^5^ from *Aspergillus niger*, which catalyzes the conversion of β-d-glucose into d-glucono-1,5-lactone (C1 oxidation) and belongs to the GMC family. Another known GMC-type carbohydrate oxidase is pyranose oxidase, which is also abundantly present in fungal proteomes and acts on monosaccharides such as d-glucose but typically oxidizes the C2 hydroxy group of hexoses.^6,7^ VAO-type carbohydrate oxidases, instead, are known to be primarily active toward oligosaccharides.^8^ The first example was described more than 30 years ago, glucooligosaccharide oxidase (GOOX)^9^ from *Acremonium strictum*. In the past several years, several more enzymes belonging to the same family have been discovered and characterized, mainly of a fungal origin: chitooligosaccharide oxidase (ChitO)^10^ from *Fusarium graminearum*, xylooligosaccharide oxidase (XylO)^11^ from *Myceliophthora thermophila*, and lactose oxidase (LaO)^12^ from *Microdochium nivale*. Recently, an enzyme with analogous features has been identified in plants.^13^ From a structural point of view, VAO-like oxidases share a common fold that is fundamentally different from that of GMC-type enzymes.^14^ The structure can be divided into two major domains: a substrate binding domain (S-domain) formed by the C-terminal part of the protein sequence and a flavin binding domain (F-domain) at the N-terminus. In general, the active site of VAO-like oxidases has a larger binding groove compared to that displayed by the GMC-oxidases,^15^ explaining their broader substrate acceptance profiles. Furthermore, enzyme engineering studies^16^ showed how the substrate specificity of these enzymes is strictly dependent on the residues that are present in the S-domain. The applications of carbohydrate oxidases are multifold. Known examples can be found in food applications^17^ and biosensors.^18^ Here, we report on the biochemical characterization, structural elucidation, and structure-inspired engineering of a recently discovered bacterial VAO-like carbohydrate oxidase.^19^ With the developed expression system, elucidated crystal structure, and established biochemical features (substrate range, kinetics, and redox potential), this newly discovered carbohydrate oxidase represents a promising biocatalyst that can be tuned for specific applications.

## Materials and Methods

### Chemicals

Ni-Sepharose 6 Fast Flow was from Cytiva, and *N*,*N*′-diacetylchitobiose was from Toronto Research Chemicals. All other chemicals were ordered from Sigma-Aldrich.

### Cloning, Transformation, Mutagenesis, and Expression

A synthetic gene encoding NagOx, codon-optimized for *Escherichia coli* with BSAI sites at the 5′ and 3′ termini (Twist Bioscience), was cloned with the Golden Gate methodology in a pBAD His-SUMO vector. For transformation, 2 μL of plasmid was added to 50 μL of *NEB10β* RbCl competent cells and incubated on ice for 30 min. Cells were then heat shocked at 42 °C for 40 s and incubated again on ice for 2 min. Then, 250 μL of prewarmed LB-SOC medium was added, and the cells were incubated for 1 h at 37 °C; 50 μL of the recovered cells was plated on LB-agar supplemented with 50 μg mL^–1^ ampicillin and incubated overnight at 37 °C. Plasmid isolation was performed, and cloning was verified through sequencing. A preinoculum of 5 mL of LB-amp (50 μg.mL^–1^) was grown overnight at 37 °C and used to inoculate 2 L baffled flasks containing 400 mL of Terrific Broth medium supplemented with 50 μg mL^–1^ ampicillin. Flasks were incubated at 37 °C until an OD_600_ of 0.6–0.8 was reached. Expression was induced with 0.02% l-arabinose, and cultures were left at 24 °C for 24 h before being harvested. Cells were harvested by centrifugation (6000 rpm, 20 min, 4 °C) and flash-frozen in liquid nitrogen.

To prepare enzyme mutants, primers were ordered from Eurofins genomics. All of the mutations were carried out using the QuickChange methodology.^20^ The PCR mix (25 μL) consisted of the following components: PfuUltra II Hotstart PCR Master Mix (12.5 μL), 1 μL of primer frw (10 μM), 1 μL of primer rev (10 μM), 1 μL of plasmid (100 ng/μL), 0.4 μL of DMSO, and MQ water up to 25 μL.

### Protein Purification

Cell pellets were resuspended in buffer A [100 mM KP_i_ and 500 mM NaCl (pH 7.5)] with a 3:1 volume (milliliters):mass (grams) ratio. Then, 0.10 mM PMSF and 1.0 mM β-mercaptoethanol were added to the lysis solution to prevent protein degradation. Cells were disrupted by sonication (5 s on, 5 s off, 70% amplitude for a total of 10 min) and then centrifuged at 11 000 rpm for 1 h. The resulting supernatant was loaded on a gravity column containing 3 mL of Ni Sepharose previously equilibrated with buffer A.

After a washing step [3 column volumes (CV)] with buffer B [50 mM KP_i_, 500 mM NaCl, and 20 mM imidazole (pH 7.5)], the protein was eluted (2 CV) with buffer C [50 mM KP_i_, 500 mM NaCl, and 500 mM imidazole (pH 7.5)]. Elution buffer was then exchanged against storage buffer [50 mM potassium phosphate buffer (pH 6.5) and 100 mM NaCl]. The concentration of the purified enzyme was measured using the extinction coefficient of 6-*S*-cysteinyl-bound FMN (ε_445_ = 11.6 mM^–1^ cm^–1^).^21,22^

### pH Optimum

Enzyme activity was analyzed by monitoring oxygen consumption at 25 °C using 1.0 μM purified enzyme [in 50 mM KP_i_ (pH 6.5)] with 5.0 mM d-glucose. The total reaction volume was 1.0 mL. Oxygen consumption was measured using an Oxygraph Plus system (Hansatech Instruments Ltd.), and the reaction was initiated by adding the enzyme. Initial rates were determined from the initial linear parts of the reaction curves.

### Thermal Stability

Due to the bicovalent protein–FAD linkage, flavin fluorescence is quenched, and thus, the ThermoFAD method could not be used to monitor enzyme unfolding.^22^ Therefore, the thermostability of the enzyme was determined using the Thermofluor assay.^23^ For these thermal unfolding assays, ∼150 μM enzyme [50 mM KP_i_ (pH 6.5)] was diluted 10-fold in various tested buffers (in duplicate) at different pH values mixed with SYPRO orange dye.^24^ The assay was performed using a RT-PCR thermocycler (CFX96 from Bio-Rad). Measurements started at 20 °C, and the temperature was increased at a rate of 1 °C/min until 95 °C.

### Steady State Kinetics

Oxidase activities on the tested carbohydrates were monitored on the basis of the formed hydrogen peroxide. Hydrogen peroxide formation was coupled to the activity of horseradish peroxidase (HRP) and chromogenic peroxidase substrates (AAP and DCHBS).^25^ Absorbance measurements were conducted on a JASCO V-660 instrument at 515 nm (ε_515_ = 26 mM^–1^ cm^–1^). Rates at different substrate concentrations were processed in GraphPad Prism and fitted using a regular Michaelis–Menten formula resulting in *K*_M_ (millimolar) and *k*_cat_ (inverse seconds) values.

### Protein Crystallization, Structural Elucidation, and Docking

After cleavage with SUMO protease, purified protein was loaded on a Superdex200 10/300 column (Cytiva) using an ÄKTA purifier. Two wavelengths (280 and 447 nm) for monitoring protein elution were used during the purification. The central fractions of the peak were pooled together and concentrated until a concentration of 12.5 mg/mL was reached on the basis of the flavin absorption peak. Different crystallization conditions were tested using a Mosquito crystallization robot (TTP LabTech, Melbourn, U.K.). Large rhomboid-shaped yellow crystals appeared under different conditions. After optimization, the best condition was obtained through sitting drop vapor diffusion using 19% PEG3350 and 0.19 M sodium nitrate; 25% glycerol was used as a cryoprotectant, and crystals were flash-frozen in liquid nitrogen and sent to ESRF for data collection. The best crystal diffracted at 1.5 Å resolution using the MASSIF-1^26^ beamline. Data were scaled using XDS. Molecular replacement was done using Phenix.^27^ Structural refinement was done using COOT^28^ and REFMAC5^29^ of the CCP4 package.^29^ The detailed statistics of the collected data set are summarized in Table 1.

**Table 1:** Data Collection and Refinement Statistics of NagOx

PDB entry	7ZZK
space group	*P*2_1_2_1_2_1_
unit cell axes (Å)	87.96, 105.01, 120.67
unit cell angles (deg)	90, 90, 90
resolution (Å)	45.00 (1.50)
*R*_merge_ (%)	7.1 (59.8)
*R*_pim_ (%)	5.3 (43.2)
CC_1/2_	0.998 (0.732)
completeness (%)	99.7 (100)
no. of unique reflections	178041 (8774)
multiplicity	2.5 (2.7)
overall *I*/σ(*I*)	12 (2.4)
no. of protein residues	972
no. of FAD molecules	2
no. of water molecules	1192
Wilson *B*-factor (Å^2^)	13.9
*R*/*R*_free_ (%)	15.1/17.8
root-mean-square deviaiton for bond lengths (Å)	0.0127
root-mean-square deviaiton for bond angles (deg)	1.77
Ramachandran outliers (%)	0.21
MolProbity score	1.38 (100th percentile)

Docking simulations were performed using the high-resolution crystal structure of NagOx. Yasara^30^ was used as a tool applying the AMBER IPQ force field with a cell of 20 Å × 20 Å × 20 Å, which included the whole active site. The experiment was conducted with 100 runs using AutoDock Vina.^31^ The results that showed a proper conformation underwent energy minimization in Yasara. The default settings were used for the computational tools.

### Redox Potential Determination

The redox potential of NagOx was determined using the xanthine/xanthine oxidase methodology.^32,33^ The reaction was performed in 50 mM KP_i_ buffer (pH 7.5) at 25 °C using a 1 mL quartz cuvette. The reaction mixture consisted of 5.0 μM benzyl viologen, 5.0 μg/mL catalase, 400 μM xanthine, a catalytic amount of xanthine oxidase, 0.5 μM 5-hydroxymethylfurfural oxidase (HMFO), and 20 mM HMF. An anaerobic condition was created by flushing the cuvette with argon for 15 min after which the HMF/HMFO system assured fully anoxic conditions. Xanthine was added to initiate the redox titration. Spectra were recorded for 1 h. Methylene blue (*E*_0_ = 11 mV) was found to a suitable dye for determining the redox potential. The *E*_M_ value was calculated by applying the Nernst equation.^32,33^

### Substrate Induced-Fit Simulations

Substrate induced-fit simulations were performed with PELE (Protein Energy Landscape Exploration), a software that combines Monte Carlo (MC) sampling with protein structure prediction algorithms.^34^ Briefly, at each MC simulation step, it executes (i) a perturbation phase, including a random translation and rotation of the ligand and a normal mode displacement of the enzyme backbone, and (ii) a relaxation phase, comprising a side chain packing optimization and overall minimization; new conformations are then accepted or rejected on the basis of the Metropolis criterion. The initial pose was prepared from the newly determined crystal structure using the Protein Preparation Wizard,^35^ where the L251R mutant was initially introduced with the builder in Maestro.^36^d-Glucose was downloaded from pubchem and prepared with LigPrep^37^ before an initial docking using Glide.^38^

## Results

### Characteristics and Stability of the Enzyme

**Figure 1:**
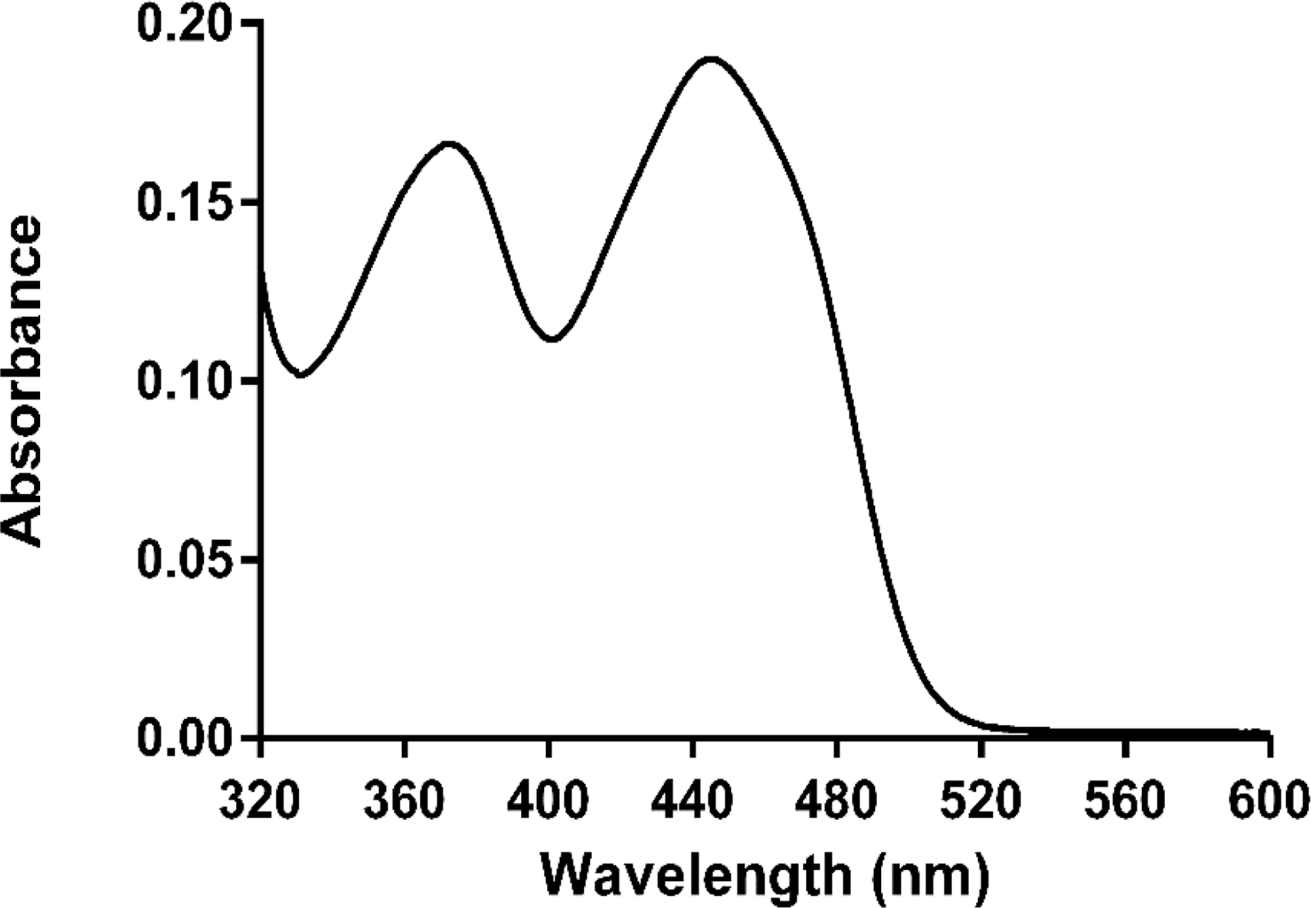
Absorbance spectrum of 20 μM NagOx [50 mM potassium phosphate buffer (pH 6.5)].

After successfully cloning the NagOx-enoding gene in a pBAD-His-SUMO vector, we overexpressed the SUMO-fused enzyme in *E. coli* NEB10-β. Approximately 40 mg of yellow-colored enzyme could be purified from a 1 L culture using a single IMAC purification step. Sodium dodecyl sulfate–polyacrylamide gel electrophoresis (SDS–PAGE) analysis showed a protein band at ∼70 kDa (Figure S1). This result matches well with the predicted molecular weight of the His-SUMO-NagOx protein (72 kDa). Incubation of the SDS–PAGE gel with 5% acetic acid for 10 min revealed under ultraviolet (UV) light the presence of a covalently bound flavin (Figure S1). The purified enzyme displayed a characteristic flavin UV–visible absorption spectrum with maxima at 447 and 375 nm (Figure 1).

**Figure 2:**
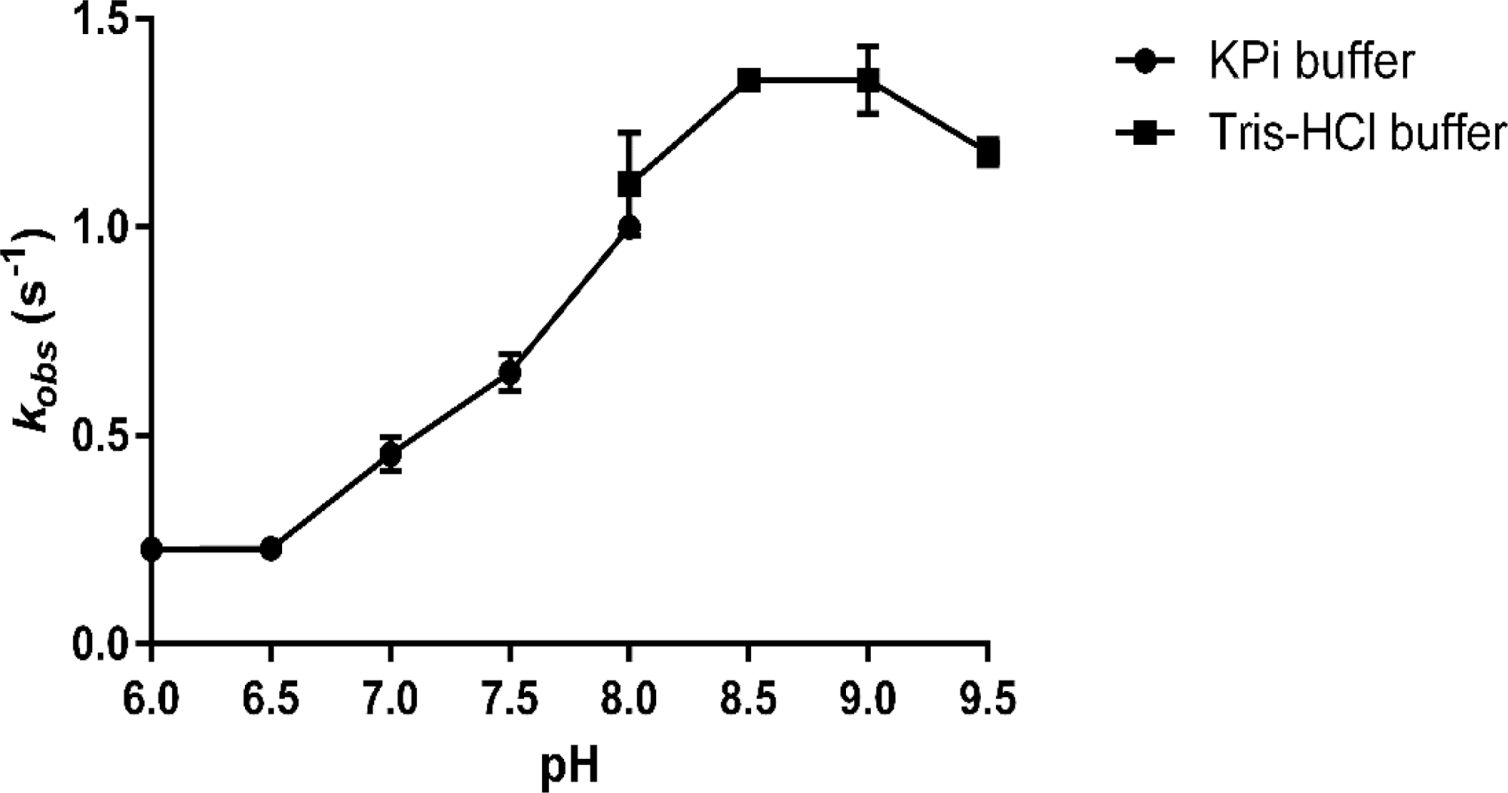
pH-dependent optimum for activity of NagOx. All reactions used 5.0 mM glucose and 1.0 μM enzyme and were performed at 25 °C. For pH 6–8, 50 mM KP_i_ buffer was used, and for pH 8–9.5, Tris-HCl was the buffer of choice.

The thermostability of NagOx was probed in different buffers with a range of pH values from 5 to 9 using the Thermofluor methodology (Table 2). The highest *T*_m_ value of 50 °C was obtained in 50 mM KP_i_ buffer (pH 6). Over a wide pH range, the enzyme displayed its highest thermostability under somewhat acidic conditions. Furthermore, we observed that additives such as NaCl (≤150 mM) and glycerol (≤5%) did not improve or reduce the stability of the enzyme. Before steady state kinetic analyses were performed, the pH optimum for the activity of the enzyme was also determined (Figure 2). In contrast to the pH optimum for stability, NagOx is more active at a relatively high pH, with an optimum at pH 8.5–9. All of our subsequent analyses were carried out in 50 mM KP_i_ buffer (pH 7.5).

**Table 2:** Thermostability of NagOx^a^

	*T*_m_ (°C)	
condition	wild-type NagOx	Leu251Arg NagOx
50 mM citrate buffer (pH 5)	43	42
50 mM citrate buffer (pH 5.5)	46	48
50 mM KP_i_ (pH 6)	50	50
50 mM KP_i_ (pH 6.5)	49	48.5
50 mM KP_i_ (pH 7)	49	50
50 mM KP_i_ (pH 7.5)	47.5	48
50 mM Tris-HCl (pH 8)	48	49
50 mM Tris-HCl (pH 8.5)	45	45.5
50 mM Tris-HCl (pH 9)	44.5	45.5

a Melting temperatures were measured using the Thermofluor method for wild-type and L251R NagOx.

With a bicovalently bound FAD as a cofactor, which was experimentally confirmed via the elucidation of the enzyme structure (vide infra), NagOx may exhibit a relatively high redox potential.^39^ To verify this, the redox potential of the FAD cofactor was determined, using the xanthine/xanthine oxidase methodology. With this protocol, a full reduction of the enzyme could be observed without any intermediate formation of a one-electron-reduced enzyme species. The redox potential of NagOx was found to be 2 mV using methylene blue (*E*_0_ = 11 mV) as a reference dye (Figure S2). While this is a relatively high redox potential for a flavoprotein, it is in the same range of redox potentials previously reported for flavoproteins containing covalent FAD that includes a histidyl linkage at the 8-methyl moiety of the flavin cofactor.^40,41^

### Substrate Screening and Steady State Kinetic Analyses

To address the substrate profile of NagOx, 37 different carbohydrates were tested (Table S1). A concentration of 10 mM was used, and activity was assessed using the HRP-based assay that detects hydrogen peroxide formation. With 1.0 μM NagOx, activity was quickly observed for *N*-acetyl-d-glucosamine, *N*-acetyl-d-galactosamine, and *N*,*N*′-diacetylchitobiose. After several minutes, peroxide formation was also observed for d-glucose, d-galactose, d-mannose, cellobiose, and maltose. These observations indicate that NagOx can act on mono- and disaccharides and seems to have a preference for N-acetylated carbohydrates. On the basis of the screening results, the steady state kinetic parameters were measured for the confirmed carbohydrate substrates mentioned above (Figure S3 and Table 3).

**Table 3:** Apparent Steady State Parameters of NagOx^a^

	wild-type NagOx			Leu251Arg NagOx		
substrate	*K*′_M_ (mM)	*k*′_cat_ (s^–1^)	*k*′_cat_/*K*′_M_ (M^–1^ s^–1^)	*K*′_M_ (mM)	*k*′_cat_ (s^–1^)	*k*′_cat_/*K*′_M_ (M^–1^ s^–1^)
*N*-acetyl-d-glucosamine	0.22 ± 0.04	140 ± 6	64 × 10^4^	23 ± 6	34 ± 3	1.5 × 10^3^
*N*-acetyl-d-galactosamine	0.13 ± 0.02	120 ± 4	93 × 10^4^	1.9 ± 0.6	4.2 ± 0.4	2.2 × 10^3^
d-glucose	92 ± 15	2.2 ± 0.1	23	6.2 ± 2.5	1.3 ± 0.2	210
d-galactose	30 ± 8	2.3 ± 0.02	77	7.7 ± 1.8	2.1 ± 0.2	270
d-mannose	36 ± 5	0.71 ± 0.03	20	14 ± 4	5.3 ± 0.5	390
*N*,*N*′-diacetylchitobiose	8.5 ± 2.2	36 ± 4	4 × 10^3^	52 ± 4	2.7 ± 0.1	52
d-cellobiose	130 ± 40	0.22 ± 0.03	2	80 ± 26	3.2 ± 0.6	40
d-maltose	>400	<0.2	0.5	22 ± 4	0.7 ± 0.1	34
maltotriose	nd	nd	–	210 ± 30	0.34 ± 0.02	1.6

a The kinetic parameters were measured at 25 °C in 50 mM KP_i_ (pH 7.5). nd indicates no activity could be measured.

The initial reaction rates were determined and could be fitted successfully in all cases using the Michaelis–Menten formula. From this analysis, it emerged that *N*-acetyl-d-glucosamine and *N*-acetyl-d-galactosamine are the preferred substrates of the enzyme with *k*_cat_ values of 120–140 s^–1^ and strikingly low submillimolar *K*_M_ values. Interestingly, the N-acetylated disaccharide *N*,*N*′-diacetylchitobiose was also found to be one of the better substrates by exhibiting a lower *k*_cat_ (36 s^–1^) and a higher *K*_M_ (8.5 mM) compared to those of the N-acetylated monosaccharide form. NagOx displays similar and relatively low catalytic efficiencies for the monosaccharides d-galactose, d-glucose, and d-mannose, which was due to relatively low *k*_cat_ (0.7–2.3 s^–1^) and high *K*_M_ (30–92 mM) values. Again, a lower catalytic efficiency was observed when compared that of the disaccharide cellobiose with d-glucose. This was mainly due to a 10-fold lower *k*_cat_. These results indicate that the *N*-acetyl moiety plays a key role in substrate recognition and that NagOx displays a better efficiency toward monosaccharides while also accepting disaccharides. The substrate acceptance profile is reminiscent of that of the fungal ChitO,^10^ yet NagOx displays a better efficiency toward monosaccharides.

### Overall Structure and Active Site of NagOx

**Figure 3:**
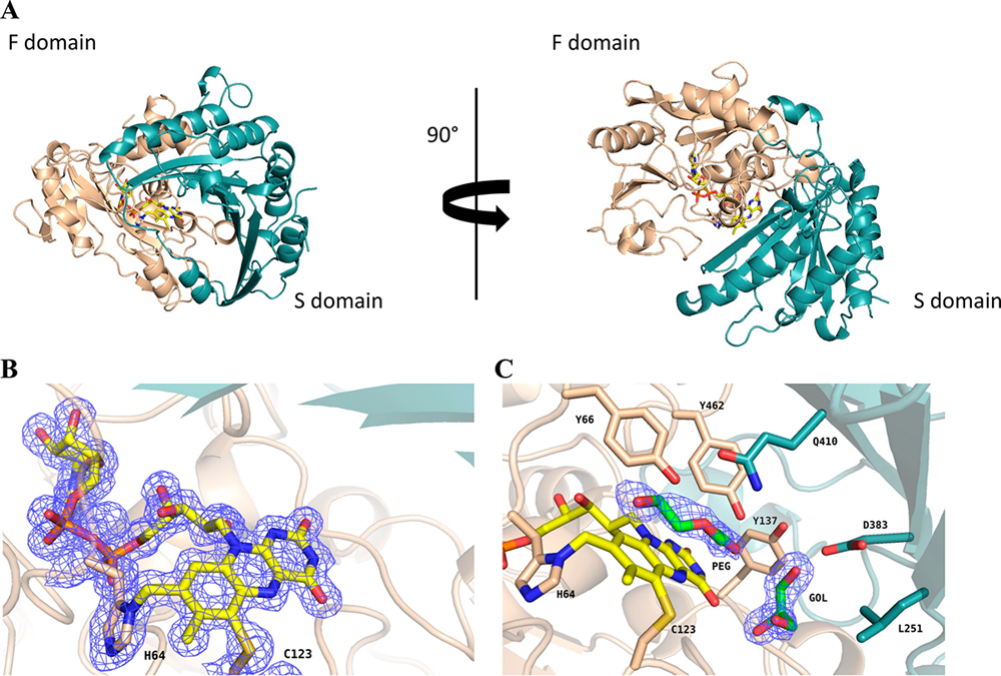
Structural analysis of NagOx. (A) The F-domain is colored wheat, the S-domain light teal, and the FAD cofactor yellow. (B) Bicovalently bound FAD via 8α-N1-histidyl and 6-S-cysteinyl linkages with a weighted 2*F*_o_ – *F*_c_ electron density map. The contour level of the map is 1.0σ. (C) Active site of NagOx. PEG and glycerol molecules are colored green, and the FAD cofactor is colored yellow.

To understand the catalytic machinery of NagOx, we set out to determine its three-dimensional structure. NagOx formed large rhomboid-shaped yellow crystals with 19% PEG3350 and 0.19 M sodium nitrate that diffracted to 1.5 Å resolution. Even though, according to size exclusion chromatograpy experiments (Figure S4), the enzyme showed a monomeric conformation in solution, two NagOx molecules were present in the asymmetric unit (Figure S5). The overall structure of NagOx is similar to those of other known flavoenzymes belonging to the VAO family. Structural alignment with ChitO [Protein Data Bank (PDB) entry 6Y0R] and GOOX (PDB entry 2AXR) resulted in root-mean-square deviations of 1.8 and 1.9 Å, respectively. Inspection of the structure revealed that the isoalloxazine moiety of the FAD cofactor is bicovalently bound at the interface between the F-domain and the S-domain. The F-domain comprises residues from the N-terminus to residue 205 and a small part of the C-terminus (residues 456–506). On the contrary, the S-domain is composed of residues 206–455 (Figure 3A). The FAD cofactor is bicovalently bound via 8α-N1-histidyl and 6-S-cysteinyl linkages with residues His64 and Cys123, respectively (Figure 3B). These characteristic bicovalent flavin–protein linkages were first observed with GOOX (His70 and Cys130) and are also present in ChitO (His64 and Cys154).^42^

The isoalloxazine ring is exposed to the solvent area at the bottom part of an open active site (Figure 3C), while the ribityl and ADP moieties of the FAD are embedded deeply in the F-domain. Other amino acids that form the active site adjacent to the redox-active isoalloxazine moiety are a string of tyrosines (Tyr66, Tyr137, Tyr345, Tyr414, Tyr459, and Tyr462) with several neighboring polar residues (Ser122, Gln381, Asp383, Gln410, and Gln412). The active site shares many common features with that of sequence-related carbohydrate oxidases such as ChitO.^42^ The same position is indeed conserved for Tyr66, Tyr462, Gln381, Asp383, and Gln410 (Table 4). Interestingly, even though the same residue is conserved for Tyr137, in NagOx the orientation is toward the isoalloxazine ring while in ChitO (Tyr168) it is pointing in the opposite direction. A short stretch of residues (292–313) is not visible in the electron density, which suggests that they form a flexible loop with a disulfide bridge at its base between Cys291 and Cys316. ChitO and GOOX do not display any flexible region or disulfide bridge in this part of the protein structure. In the active site of NagOx, a molecule of ethylene glycol and glycerol are bound, providing a hint about how NagOx may interact with its carbohydrate substrate. The ethylene glycol C1 atom is 3.0 Å above the N5 atom of the flavin. The O1 atom interacts with Tyr462 OH (3.1 Å) and Gln410 NE2 (3.2 Å). This result is in line with what was observed in the previously elucidated structures of XylO and GOOX. In both cases, a Tyr residue in the analogous position was described to interact with O1 of the bound carbohydrate ligand and possibly act as a catalytic base. The molecule of glycerol is instead placed in a secondary pocket. C1 is placed 3.6 Å from Leu251; O3 interacts with Asp383 OD2 (2.6 Å) and Tyr137 OH (3.6 Å), and O2 is placed 3.4 Å from Ser122 OG.

**Table 4:** Comparison of the Active Sites of NagOx, ChitO, and GOOX^a^

NagOx	ChitO	GOOX
**Tyr66**	**Tyr96**	**Tyr72**
**Tyr137**	**Tyr168**	**Tyr144**
**Tyr345**	Ala341	Ala318
**Tyr414**	Ser410	Ser388
**Tyr459**	**Tyr444**	**Tyr426**
**Tyr462**	**Tyr447**	**Tyr429**
**Ser122**	Thr153	Thr129
**Gln381**	**Gln375**	**Gln353**
**Asp383**	**Asp377**	**Asp355**
**Gln410**	**Gln406**	**Gln384**
**Gln412**	Tyr408	Tyr386

a Conserved residues are shown in bold.

### Engineering NagOx toward Activity on Non-N-acetylated Carbohydrates

**Figure 4:**
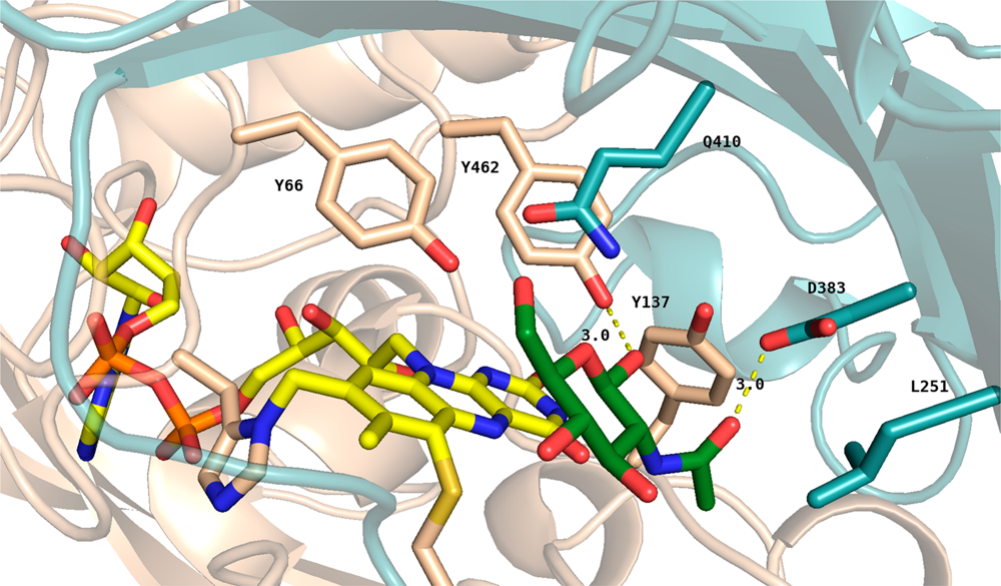
Docked *N*-acetyl-d-glucosamine in the active site of NagOx. *N*-Acetyl-d-glucosamine is colored dark green, and the FAD cofactor yellow. Distances are expressed in angstroms.

Despite several soaking attempts with N-acetylated compounds, no crystal structure in complex with a carbohydrate was obtained. Instead, an *in silico* analysis was performed to understand which residues are involved in substrate binding. From our previous study of NagOx, one could conclude that oxidation occurs at C1 of NagOx carbohydrate substrates.^19^ This provides input on how the substrate should be positioned with respect to N5 of the flavin cofactor. *N*-Acetyl-d-glucosamine was docked into the structure, which revealed a binding pose in which the *N*-acetyl moiety can occupy the secondary hydrophobic pocket of the active site (Figure 4).

The observed binding mode of the acetyl moiety is nicely in line with the bound glycerol. The bound PEG molecule occupies the locus in which the hexose moiety of *N*-acetyl-d-glucosamine was docked in the crystal structure. The suggested binding is in line with what was observed for ChitO, where a similar pocket can accommodate *N*-acyl moieties attached at C2.^42^ The secondary pocket in NagOx is formed by residues Ser122, Tyr137, Leu139, Leu251, Val334, Gln381, Asp383, Tyr459, and Tyr462. While for ChitO, Gln268 was found to play a key role in accommodating the *N*-acetyl moiety, NagOx has a leucine (Leu251) in the analogous position. The hydroxyl moiety at C1 of the sugar points toward Tyr462 (3.0 Å distance), which may act as the base to trigger or enable hydride transfer from the substrate to N5 of the FAD cofactor (vide supra). Such a mechanism is also in line with the distance from N5 to C1 of the docked substrate (3.4 Å).^43^ The observed substrate binding mode was used as the basis for an enzyme engineering effort. To alter the substrate acceptance of NagOx with respect to non-N-acetylated monosaccharides (such as d-glucose and d-galactose) and disaccharides (such as cellobiose and maltose), an enzyme variant was generated in which Leu251 was replaced. Because the replacement of Gln268 with an arginine in ChitO improved the activity toward glucooligosaccharides,^16^ we prepared the Leu251Arg NagOx mutant to probe the effect on substrate acceptance in this bacterial oxidase. The Leu251Arg mutant could be overexpressed and purified with yields similar to those of wild-type NagOx and displayed similar thermostability (Table 2). Next, a steady state kinetic analysis was carried out (Table 3). Compared to that of wild-type NagOx, the activity toward *N*-acetyl-d-glucosamine and *N*-acetyl-d-galactosamine was drastically reduced. The catalytic efficiency decreased ∼400-fold for both N-acetylated monosaccharides. The activity toward *N*,*N*′-diacetylchitobiose was also reduced with a decrease in catalytic efficiency by 2 orders of magnitude. The decreased catalytic performance on these substrates was caused by lower *k*_cat_ and *K*_M_ values. These data confirm the role of Leu251 in positioning these N-acetylated carbohydrates in the active site. Interestingly, the activity toward non-N-acetylated hexoses was improved. For d-glucose and d-galactose, while the *k*_cat_ values were hardly affected, the *K*_M_ values decreased to values of <10 mM, resulting in increases in catalytic efficiency of 9-fold for d-glucose and 3.5-fold for d-galactose. In fact, the specificity of the Leu251Arg NagOx mutant for d-glucose (*K*_M_ = 6.2 mM) is significantly higher than that of the widely applied and commercially available glucose oxidase from *A. niger* (*K*_M_ = 26 mM).^2^ The catalytic efficiencies for d-mannose and cellulose improved 20-fold. Interestingly, the largest beneficial effect on catalytic performance was found for the disaccharide maltose (70-fold improvement in catalytic efficiency). Testing maltotriose revealed a similar trend. While no significant activity could be observed with wild-type NagOx, the Leu251Arg mutation introduced activity for this trisaccharide (*K*_M_ = 210 mM, and *k*_cat_ = 0.34 s^–1^).

### Modeling d-Glucose Binding in the L251R Variant

To understand the decrease in *K*_M_ for d-glucose in the L251R variant, we again performed simulations. Moreover, to address potential local conformational changes, this time we turned to induced-fit calculations with the PELE software, which is capable of quickly mapping the conformational changes associated with protein–ligand interactions.^44^Figure S6a shows the d-glucose–NagOx interaction energy profiles along the (active site) induced-fit simulation for the wild type and the L251R mutant. Clearly, we observe significantly lower interaction energies for the engineered variant at short catalytic distances, indicating better substrate binding in terms of energies and in achieving catalytic poses. Inspecting the best enzyme–substrate pose for L251R, we observed a good proton abstraction distance, 1.97 Å, which seemed to be partially driven by direct interaction with Arg251 (Figure S6b).

## Discussion

Flavoprotein oxidases acting on carbohydrates are versatile and valued biocatalysts typically displaying high substrate specificity and regioselectivity. Nevertheless, almost all carbohydrate oxidases characterized so far are of eukaryotic origin, and therefore, the expression in a bacterial host is often challenging, hampering further enzyme engineering strategies and/or development of applications. The recent identification of a carbohydrate oxidase (NagOx) from the bacterium *Ralstonia solanacearum* with the same regioselectivity^19^ as previously discovered fungal analogues inspired us to further investigate this enzyme. NagOx belongs to the VAO-type flavoprotein oxidase superfamily and can be overexpressed in *E. coli* with a yield of 40 mg/L after one affinity chromatography purification step. We observed that the enzyme is most stable under somewhat acidic conditions, while it is most active at relatively high pH. Steady state kinetic analyses revealed the highest activity and specificity toward N-acetylated monosaccharides (*N*-acetyl-d-glucosamine and *N*-acetyl-d-galactosamine) and the disaccharide *N*-diacetylchitobiose. Only a minor activity was registered for non-N-acetylated monosaccharides (d-glucose, d-galactose, and d-mannose) and disaccharides (maltose and cellobiose).

To identify the structural features that dictate the substrate profile and to tune the substrate scope by structure-based enzyme engineering, the crystal structure of NagOx was determined. Inspection of the active site confirmed that the FAD is bicovalently bound via 8α-N1-histidyl and 6-S-cysteinyl linkages with residues His64 and Cys123. The crystal structure also revealed a clear solvent-exposed binding pocket in front of the isoalloxazine moiety of the flavin cofactor that allows binding of mono- and oligosaccharides. We also identified a secondary binding pocket similar to that present in ChitO,^38^ with residue Leu251 playing a key role in accommodating the *N*-acetyl moiety. On the basis of these insights, we designed and prepared a specific enzyme variant (L251R) that displays a more relaxed substrate preference. The L251R variant had an improved catalytic efficiency toward all non-acetylated monosaccharides and disaccharides. Docking and modeling glucose binding confirmed that the introduced arginine promotes productive binding of glucose (Figure S5b). Furthermore, the activity toward the trisaccharide maltotriose was introduced. Due to its advantage of being functionally expressed in a good yield in a bacterial host, NagOx is a promising alternative for engineering carbohydrate oxidases for selective oxidation of monosaccharides and disaccharides.

## Supporting Information Available

SDS–PAGE gel pictures (Figure S1), redox determination data (Figure S2), plots with steady state kinetic data (Figure S3), size exclusion chromatography profile (Figure S4), dimeric structure of NagOx (Figure S5), and screening of substrates (Table S1) (PDF)

## Accession Codes

NagOx, A3RXB7.
