# Supplementary material for: Structural Elucidation and Engineering of a Bacterial Carbohydrate Oxidase
Source: Biochemistry. 2022 Jul 26;62(2):429–36. doi: 10.1021/acs.biochem.2c00307 (PMC9850908; doi:10.1021/acs.biochem.2c00307)
Supplement: Supplementary file 1 — bi2c00307_si_001.pdf [file bi2c00307_si_001.pdf]

Structure elucidation and engineering of a bacterial carbohydrate oxidase

Alessandro Boverio[1,2], Wahyu S. Widodo[1], Lars L. Santema[1], Henriëtte J. Rozeboom[1], Ruite Xiang[3], Víctor Guallar[3], Andrea Mattevi[2], Marco W. Fraaije[1*]

[1] *Molecular Enzymology, Groningen Biomolecular Sciences and Biotechnology Institute, University of Groningen, 9747AG Groningen, The Netherlands*

[2] *Department of Biology and Biotechnology, University of Pavia, via Ferrata 9, 27100 Pavia, Italy*

[3] *Electronic and atomic protein modelling group, Barcelona Supercomputing Center, E-08034 Barcelona, Spain.*

Supporting material

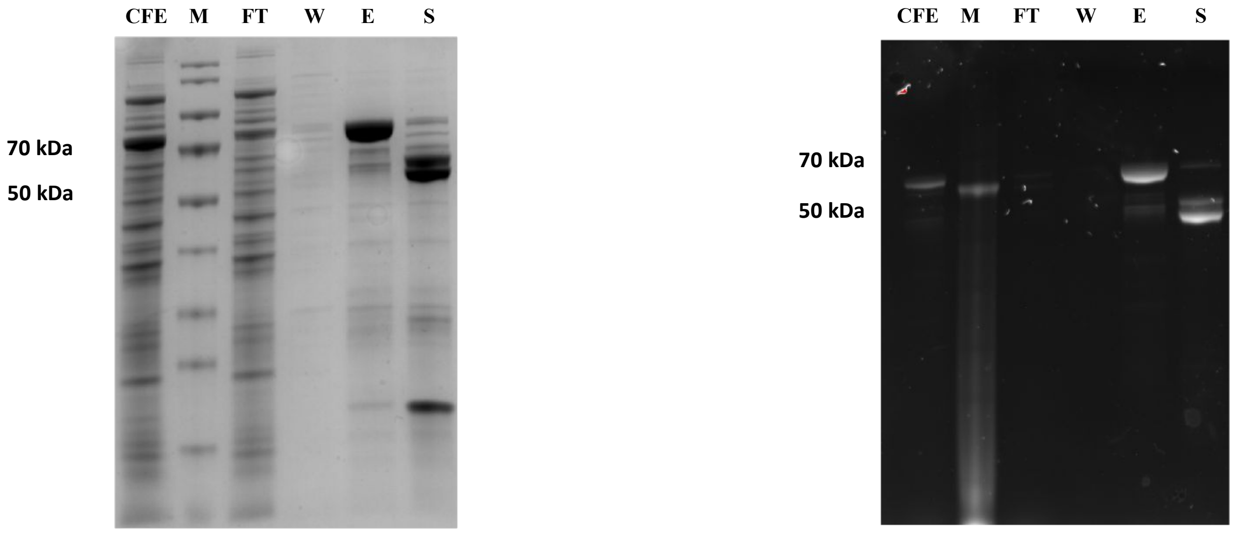

Figure S1. SDS-PAGE gels of a purification of NagOx. The left image shows different purification steps upon protein staining, the right image is of the same gel but monitored by fluorescence (of covalent FAD). CFE: cell free extract; M: molecular weight markers; FT: flow-through; W: washing step; E: elution step; S: result after SUMO cleavage using SUMO protease.

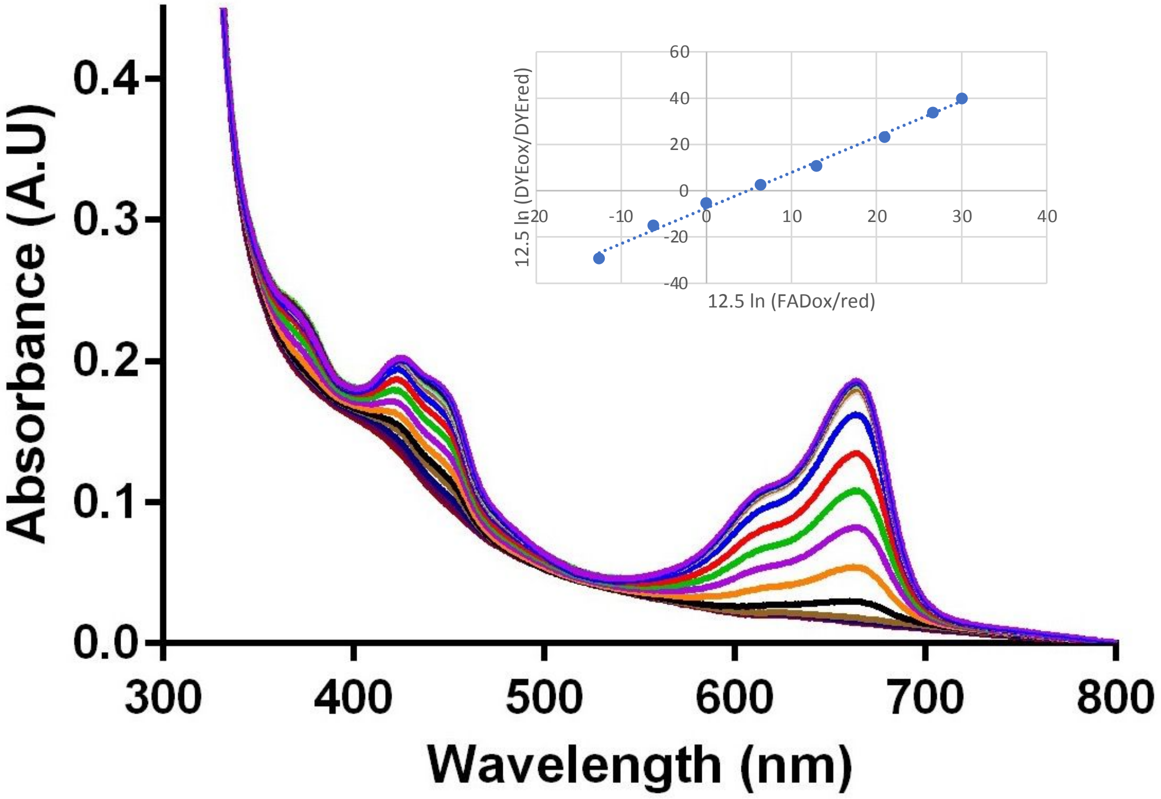

Figure S2. Determination of the midpoint redox potential (E_m) of NagOx using the xanthine/xanthine oxidase methods and methylene blue as dye. The inset shows a plot in which the $\log[(Ox_{dye})/(Red_{dye})]$ is on the Y-axis, and $\log[(Ox_{enzyme})/(Red_{enzyme})]$ is on the X-axis. The redox potential of the enzyme was found to be +2 mV (the slope of the fitted data was 1.5).

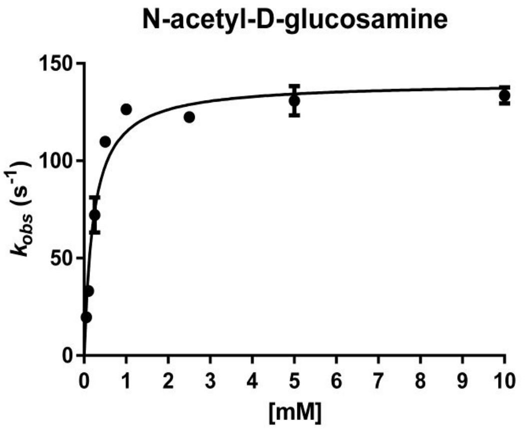
N-acetyl-D-glucosamine
k_{obs} (s^{-1})
0
50
100
150
0 1 2 3 4 5 6 7 8 9 10
[mM]

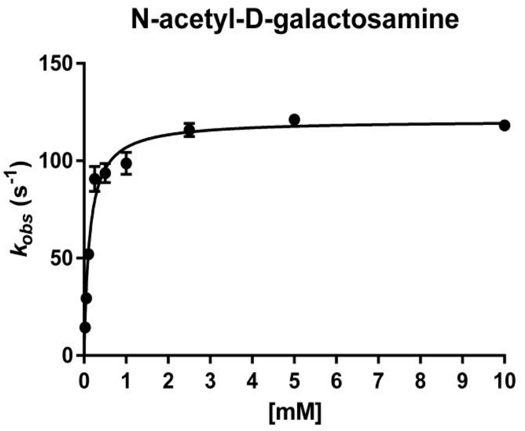
N-acetyl-D-galactosamine
k_{obs} (s^{-1})
0
50
100
150
0 1 2 3 4 5 6 7 8 9 10
[mM]

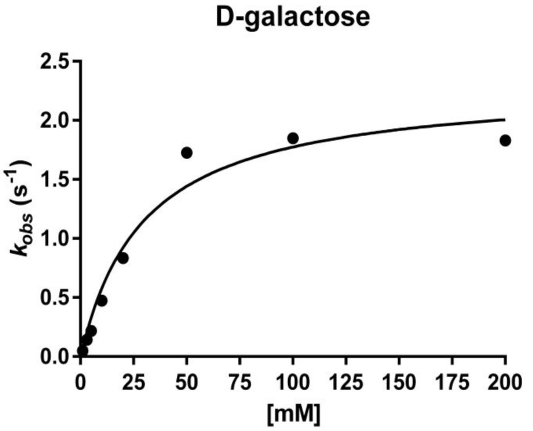
D-galactose
k_{obs} (s^{-1})
0.0
0.5
1.0
1.5
2.0
2.5
0 25 50 75 100 125 150 175 200
[mM]

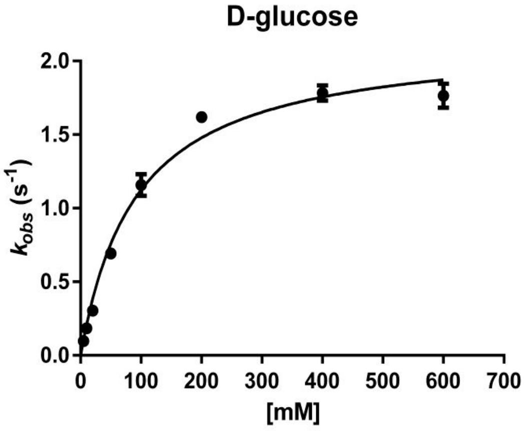
D-glucose
k_{obs} (s^{-1})
0.0
0.5
1.0
1.5
2.0
0 100 200 300 400 500 600 700
[mM]

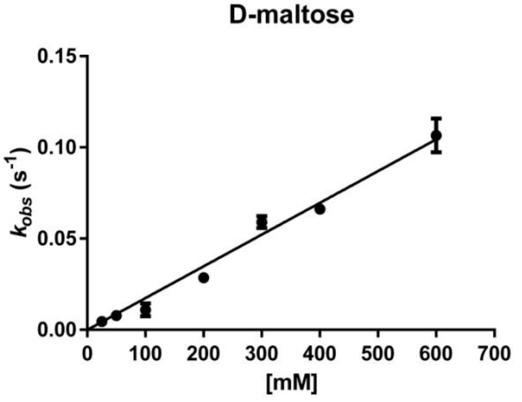
D-maltose
k_{obs} (s^{-1})
0.00
0.05
0.10
0.15
0 100 200 300 400 500 600 700
[mM]

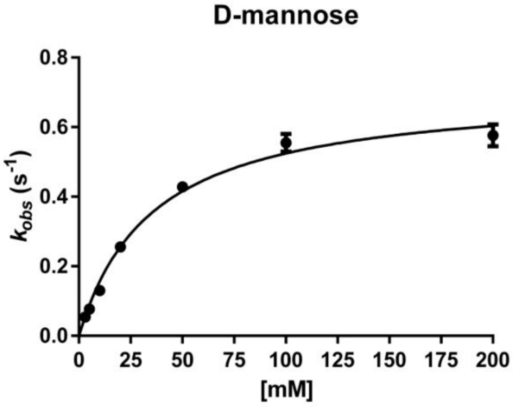
D-mannose
k_{obs} (s^{-1})
0.0
0.2
0.4
0.6
0.8
0 25 50 75 100 125 150 175 200
[mM]

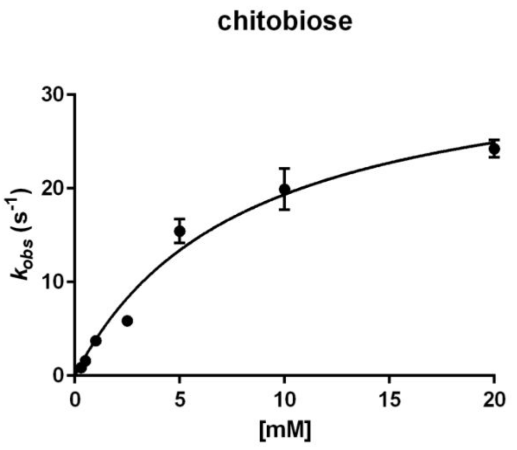
chitobiose
30
20
10
0
k_{obs} (s^{-1})
0
5
10
15
20
[mM]

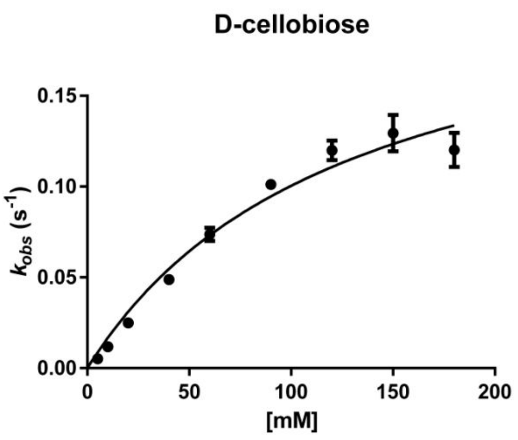
D-cellobiose
0.15
0.10
0.05
0.00
k_{obs} (s^{-1})
0
50
100
150
200
[mM]

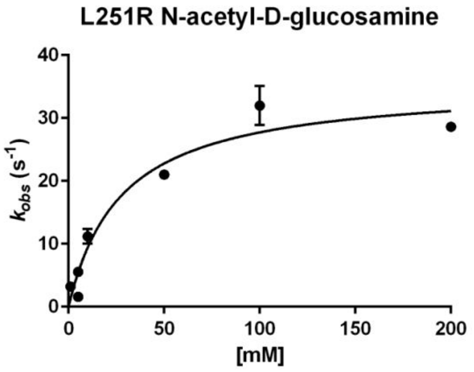
L251R N-acetyl-D-glucosamine
40
30
20
10
0
k_{obs} (s^{-1})
0
50
100
150
200
[mM]

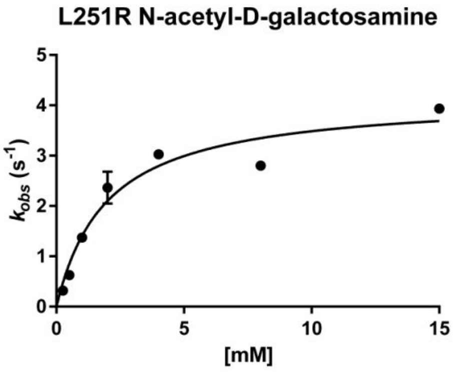
L251R N-acetyl-D-galactosamine
5
4
3
2
1
0
k_{obs} (s^{-1})
0
5
10
15
[mM]

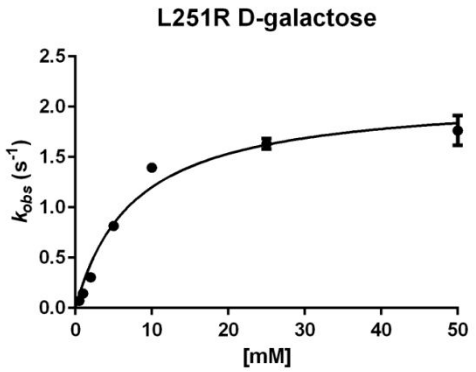
L251R D-galactose
2.5
2.0
1.5
1.0
0.5
0.0
k_{obs} (s^{-1})
0
10
20
30
40
50
[mM]

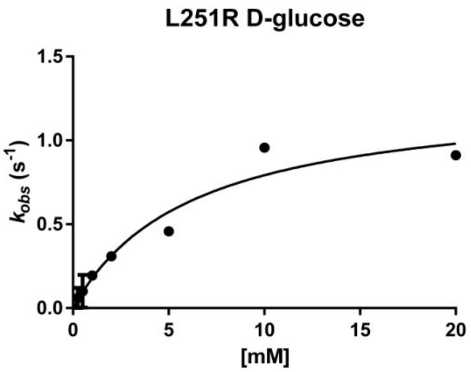
L251R D-glucose
1.5
1.0
0.5
0.0
k_{obs} (s^{-1})
0
5
10
15
20
[mM]

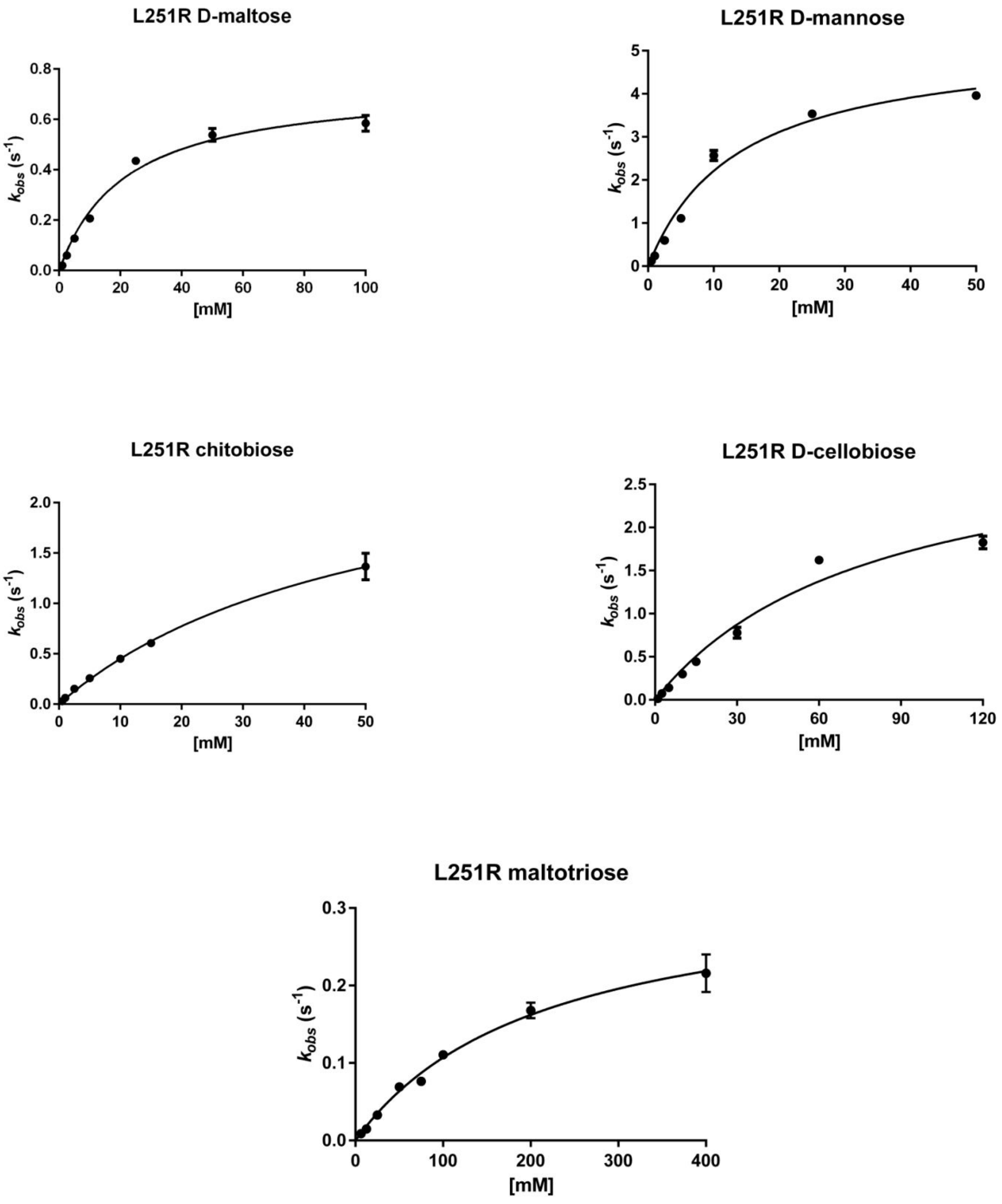

Figure S3. Michaelis-Menten fit of the values obtained testing NagOx WT and L251R with different carbohydrates. All points were taken in triplicates.

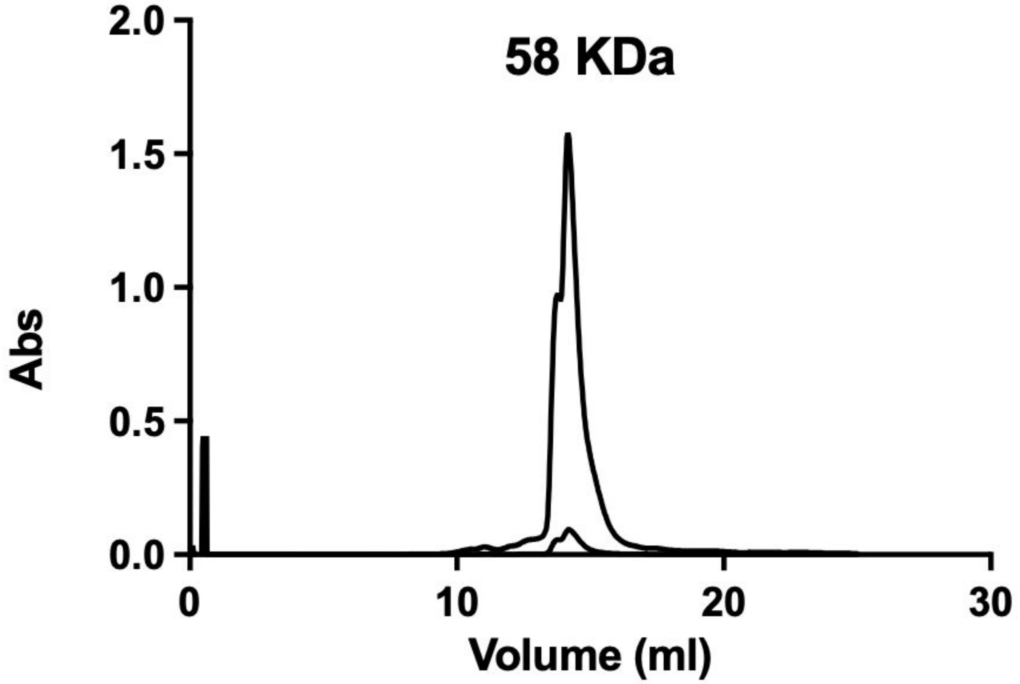

Figure S4. SEC profile of NagOx using a Superdex 200 10/300 column using 50 mM KPi buffer pH 6.5. Two different wavelengths (280 nm [high absorbance] and 447 nm) were used to monitor the protein.

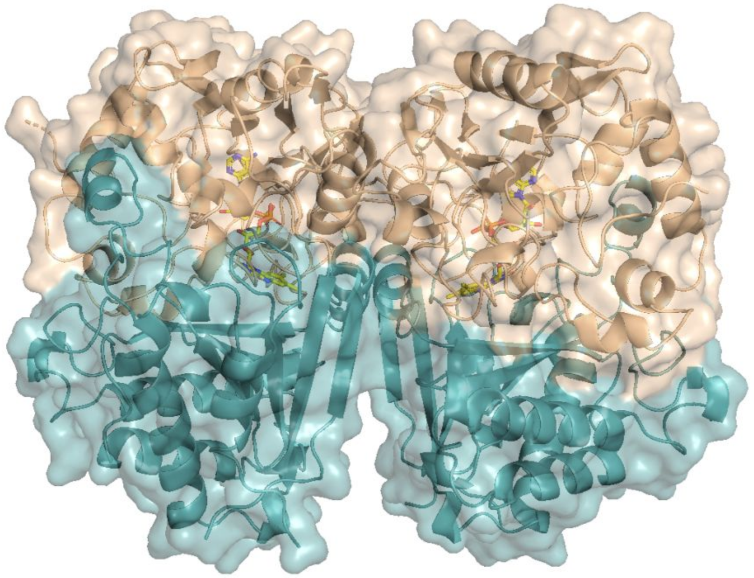

Figure S5. Structure of the NagOx dimer. 2 molecules were present in the asymmetric unit resulting in a limited entrance in the active site. F-domain is represented in cyan; S-domain is represented in green. FAD molecules are in yellow

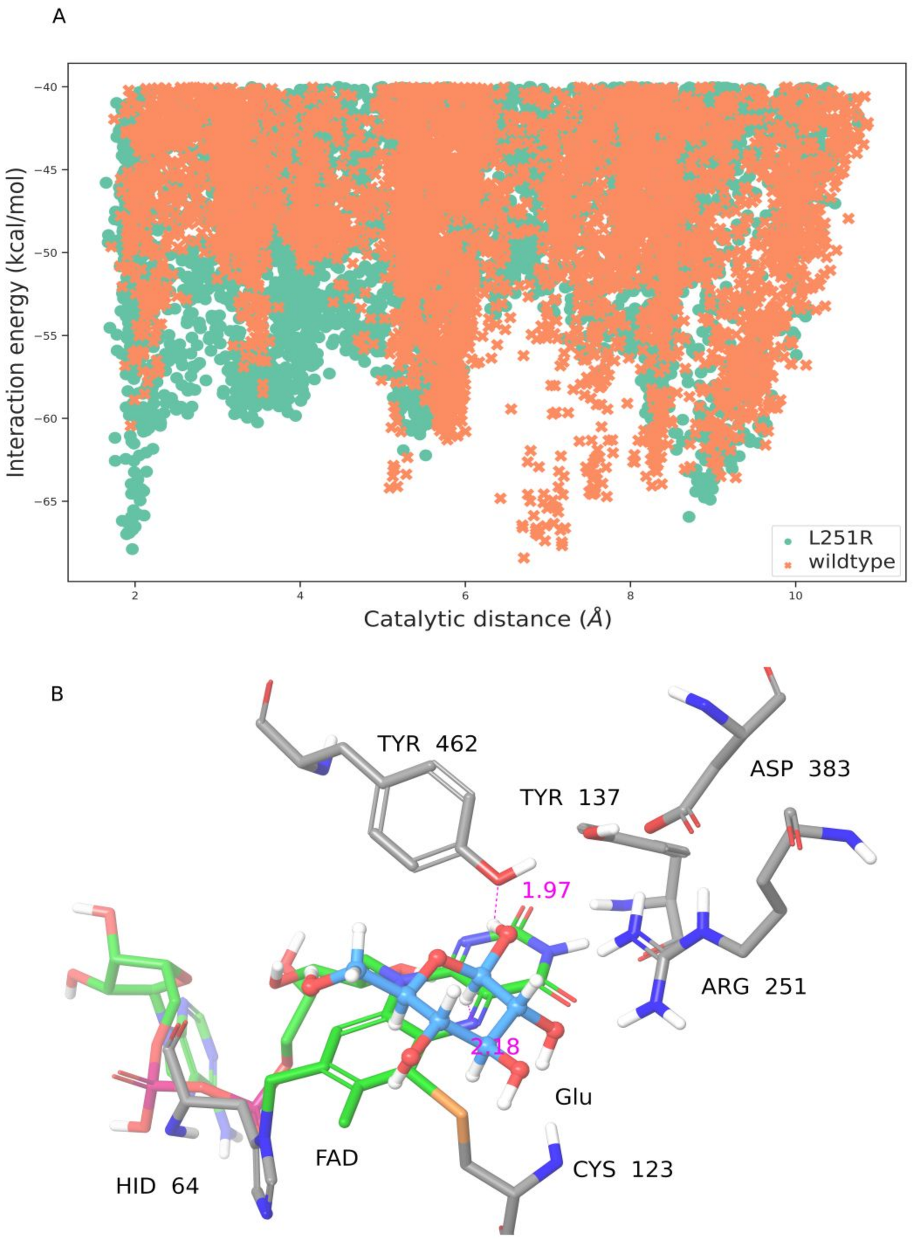

Figure S6. A) PELE induce-fit energy profiles for D-glucose in the WT and the L251R variant. B) Structure of the best pose as found for L251R, where we underline the catalytic distance used to monitor the energy profiles in pink.

Table S1. Substrate screening NagOx. Activity screen of NagOx using the HRP based method assay. In total 39 different substrates were tested.

Substrate	***Activity***
Monosaccharides	
D-Glucose	✓
D-Galactose	✓
D-Mannose	✓
D-Xylose	X
L-Arabinose	X
N-acetyl glucosamine	✓
N-Acetyl galactosamine	✓
Rhamnose	X
D-Ribose	X
L-Fucose	X
Disaccharides	
D-Lyxose	X
D-xylose	X
Chitobiose	✓
Oligosaccharides	X
D-Cellobiose	✓
Lactose	X
Xylobiose	X
D-Trehalose	X
Sucrose	X
D-Maltose	✓
Melibiose	X
Sugar alcohols	
Xylitol	X
Ribitol	X
Sorbitol	X
D-Arabitol	X
Galactitol (dulcitol)	X
Vanillyl alcohol	X
Cyclic sugars-aromatic alcohols	
β-cyclodextrin (cycloheptaamylose)	X
Syringic acid	X
Ferulic acid	X
Caffeic acid	X
Sinapic acid	X
Coumaric acid	X
D-galacturonic acid	X
Polysaccharides	
Xylan	X
Arabinan	X
Lactones	
D-gluconic acid δ lactone	X
L-gulonic acid δ lactone	X
D-arabino-1,4 lactone	X
L-fucono-1,4 lactone	X
